# Case Report: A 72-Year-Old Woman With Progressive Motor Weakness, Dry Eyes and High Levels of Serum Neurofilament Light Chain

**DOI:** 10.3389/fneur.2022.889894

**Published:** 2022-07-11

**Authors:** Maria Janina Wendebourg, Jens Kuhle, Martin Hardmeier

**Affiliations:** ^1^Department of Neurology, University Hospital Basel, Basel, Switzerland; ^2^Department of Clinical Research, University of Basel, Basel, Switzerland

**Keywords:** case report, neuromuscular disorders, peripheral neuropathies, C9*orf* 72, serum neurofilament light chain (sNfL), Amyotrophic Lateral Sclerosis, Sjögren's disease

## Abstract

**Background:**

Diagnosis of Amyotrophic Lateral Sclerosis (ALS) is challenging as initial presentations are various and diagnostic biomarkers are lacking. The diagnosis relies on the presence of both upper and lower motor neuron signs and thorough exclusion of differential diagnoses, particularly as receiving an ALS diagnosis has major implications for the patient. Sjögren's syndrome may mimic peripheral ALS phenotypes and should be considered in the work-up.

**Case:**

A 72-year-old female presented with a mono-neuropathy of the right leg and a complaint of dry eyes and mouth. Initial diagnostic work-up confirmed a regional sensorimotor neuropathy and a Sjögren's syndrome; a causal relationship was assumed. However, motor symptoms spread progressively despite immunosuppressive treatment, eventually including both legs, both arms and the diaphragm. Clinically, unequivocal central signs were lacking, but further along in the disease course, the atrophy pattern followed a split phenotype and deep tendon reflexes were preserved. Nerve biopsy did not show vasculitic infiltration; however, serum neurofilament light chain (sNfL) concentrations were and remained persistently highly elevated despite immunosuppressive treatment. Electrodiagnostic re-evaluation confirmed denervation in 3 regions. A diagnosis of familial ALS was finally confirmed by a C9*orf* 72 repeat expansion. Stationary sensory symptoms were best explained by a neuropathy associated with concomitant Sjögren's syndrome

**Discussion:**

Our instructive case shows the difficulties of diagnosing ALS in the setting of a peripheral symptom onset and a concurrent but unrelated condition also causing neuropathy. Such cases require high clinical vigilance and readiness to reappraise diagnostic findings if the disease course deviates from expectation. Recently proposed simplified diagnostic criteria, genetic testing and body fluid biomarkers such as sNfL may facilitate the diagnostic process and lead to an earlier diagnosis of ALS.

## Introduction

Amyotrophic Lateral Sclerosis (ALS) is a fatal neurodegenerative disease characterized by the degeneration of upper (UMN) and lower motor neurons (LMN), leading to the combination of muscle weakness, atrophy, and spasticity with exaggerated deep tendon reflexes (1), and may include frontal lobe dysfunction. Its prevalence is 2.6–3.0/100,000 in populations of European descent. Death occurs mainly from respiratory failure. The mean time of survival is 2–3 years, but in some cases, patients live for more than 10 years. There are considerable differences in symptom presentation and clinical course. While the majority of cases present with a limb-onset spinal type, others present with bulbar symptoms such as dysarthria and dysphagia, or primarily with signs of UMN dysfunction (1).

Diagnosis of ALS is still mostly clinically based on the El Escorial criteria and their revisions (2, 3), and, more recently, on the Gold Coast criteria (4) (see Table 1). The latter require progressive motor impairment, the clinical presence of UMN and LMN signs or LMN signs in two regions as well as the thorough exclusion of ALS mimics.

**Table 1:** Comparison of the El Escorial (2), Awaji (3), and Gold Coast criteria (4) for Amyotrophic Lateral Sclerosis.

	**El Escorial criteria (2)**	**Awaji criteria (3)**	**Gold Coast criteria (4)**
	Progressive spread of symptoms or signs **AND**		Progressive motor impairment **AND**
Possible ALS	UMN and LMN signs in 1 region	UMN and LMN signs in 1 region	UMN and LMN signs in at least 1 region or LMN signs in at least 2 regions
Probable ALS	UMN and LMN signs in 2 regions	UMN and LMN signs in 2 regions	
Probable ALS (laboratory supported)	UMN signs in 1 region + electrophysiologic evidence of LMN signs in 2 regions		
Definite ALS	UMN and LMN signs in 3 regions	UMN and LMN signs in 3 regions	
	Mandatory: Exclusion of other etiologies explaining clinical symptoms		

*In Awaji criteria, ENMG can substitute clinical evidence of LMN involvement on each diagnostic level. Regions are defined as: bulbar, cervical, thoracic, and lumbar*.

*UMN, upper motor neuron; LMN, lower motor neuron*.

Diagnostic biomarkers other than electromyoneurography (ENMG) to objectify LMN (3) are currently not part of diagnostic criteria. However, UMN dysfunction may be shown by increased cortical excitability as determined by transcranial magnetic stimulation (5, 6), particularly in early stages of the disease, or on imaging, where atrophy of the precentral gyri and alterations of the cortico-spinal tract in various MRI measures has been reported (7, 8).

Furthermore, several studies have recently shown that neurofilament light (NfL) chain levels are highly increased in CSF and serum of patients with ALS and they have been repeatedly shown to harbor independent prognostic information besides potentially qualifying as a diagnostic biomarker (9–11). However, NfL is not disease specific but quantitatively captures neuro-axonal damage in inflammatory, neurodegenerative, vascular and traumatic brain diseases (12), as well as in peripheral disorders such as vasculitic and other polyneuropathies (13, 14).

Genetic analyses may assist diagnosis as about 5%−15% of ALS cases are familial (fALS) with autosomal dominant inheritance. The most prevalent mutations concern a repeat expansion of the C9*orf* 72 gene (Chromosome 9 open reading frame 72; 30−40%), the SOD1 gene (superoxide dismutase 1, 10−20%), the TDP-43 gene (transactive response DNA-binding protein 43; 5%), and the FUS gene (fused-in sarcoma, 5%) (15). A C9*orf* 72 repeat expansion is found in 7−19% of apparent sporadic ALS cases.

Differential diagnoses comprise, among others, multifocal motor neuropathy, Kennedy disease, lead intoxication and spinal stenosis. A recent study highlighted Sjögren's motor neuropathy as a further entity to consider (16). Mimics may cause up to 10% false ALS diagnosis (17), but likewise, ALS may not be diagnosed in first place due to the presence of other diseases.

We present the case of a patient with sicca symptoms and a progressive, predominantly flaccid, motor paralysis with mild sensory symptoms who was diagnosed with Sjögren's syndrome and associated neuropathy but eventually turned out to be a familial ALS with a C9*orf* 72 repeat expansion.

Our case highlights the challenges in diagnosing ALS in the presence of a treatable co-existing disease and we discuss the differential diagnostic considerations during progression and evolution of symptoms as well as the value of biomarkers.

## Case Description

**Figure 1:**
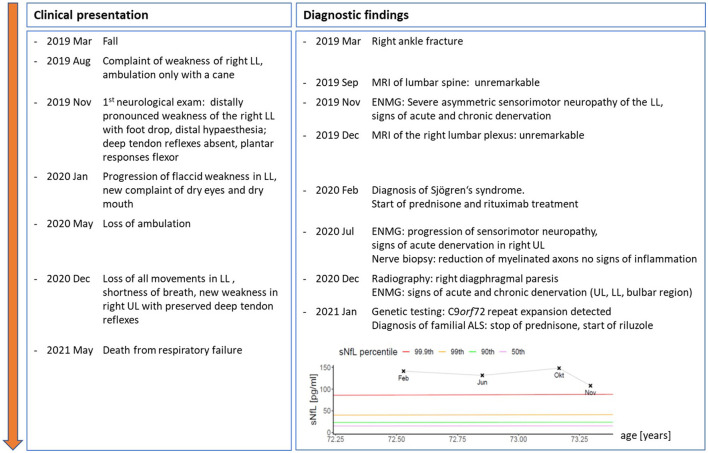
Time course of clinical symptoms and signs as well as results from ancillary examinations and therapeutic interventions from symptom onset to death. Inserted graph shows serial serum neurofilament light chain levels (sNFL) from Feb to Nov 2020, coloured lines give age- and BMI-adjusted percentiles of reference population (18). UL, upper limbs; LL, lower limbs; ENMG, electroneuromyography.

A 70-year-old woman presented with persistent weakness of the right leg after a fall and fracture of the right ankle 10 months before. She had no pain but complained of dry eyes and a dry mouth. Personal history was remarkable for a colon carcinoma operated 30 years ago, a metabolic syndrome and endoprosthesis of both knees. Only in the later follow-up, the patient reported that her mother had died from bulbar ALS. There was no known history of autoimmune disease in the family. The time course of both, clinical evolution and results of ancillary examinations are depicted in Figure 1.

At first outpatient consultation, we saw an overweight woman (BMI 43 kg/m^2^) in otherwise good health. The cranial nerve examination was unremarkable. Upper limbs and the left leg were normal including preserved deep tendon reflexes and reported normal sensory function. The right leg showed a distally pronounced weakness (right/left, Medical Research Council (MRC) grades): hip flexion 2/5, hip extension 4/5, hip adduction 5/5, hip abduction 2/5, knee flexion 2/5, knee extension 4/5, foot dorsal extension 0/5, foot plantar flexion 3/5. Deep tendon reflexes were absent in the right leg and plantar responses were flexor bilaterally. We observed no fasciculations. The right lateral lower leg and dorsal foot showed a marked sensory deficit while vibration sense was only minimally impaired (7/8 at both halluces).

Neurographies in the right leg were abnormal: The peroneal compound muscle action potential (CMAP) was absent, the tibial CMAP and the sural sensory nerve action potential (SNAP) showed markedly reduced amplitudes without conduction block (CB). The left leg showed a slightly decreased peroneal CMAP and otherwise normal neurographies. Myography showed severe signs of acute denervation in the right anterior tibial, gastrocnemius, and rectus femoris muscle with fasciculations, positive sharp waves and fibrillation potentials. Plain MRI of the lumbar spine showed no abnormalities. An asymmetric sensorimotor axonal neuropathy was diagnosed.

Further work-up included laboratory screening for treatable causes of neuropathy, a spinal tap and measurement of sNfL levels. Anti-SSA/ro antibodies (55 U/ml; cut-off <10 U/ml), anti-GM1 (116%; cut-off 50%), anti-GD1a (130%; cut-off 50%) and anti-GD1b (98%; cut-off 50%) were elevated. Anti-SSB/la antibodies, cryoglobulines, blood sedimentation rate and the remainder of the laboratory screening including serum creatine kinase (CK) levels were normal. Cerebrospinal fluid (CSF) analysis showed a normal cell count, negative CSF-specific oligoclonal bands (OKBs) and only slightly elevated total protein (515 g/L, reference: 150–500 mg/L; quotient albumin CSF/serum: normal). Serum NfL levels were markedly elevated [141.5 pg/ml; >99.9th percentile (18)] indicating severe ongoing neuro-axonal injury. Nerve ultrasound had limited validity due to the patient's obesity but showed no detectable abnormalities.

The clinical suspicion of Sjögren's disease was supported by a positive Schirmer test and sialometry showing pathologic reductions of tear and saliva production. On lip biopsy, interstitial fibrosis and lymphatic invasion of small saliva glands was demonstrated and a diagnosis of Sjögren's disease was made according to the ACR criteria (19).

At this stage, the neuropathy was considered to be attributable to Sjögren's disease. Prednisone (80 mg/day, with tapering) as well as Rituximab treatment was established. Treatment was expected to improve or at a least stabilize motor (and sensory) function.

Contrarily, the weakness progressed further over the course of the next months, spreading to the left leg, rendering the patient wheelchair bound. Moreover, she began to complain of shortness of breath. Upon re-admission, the clinical examination was normal for cognition and cranial nerve function. In the upper limbs, we found focal atrophies of the right first dorsal interosseous (FDI), abductor pollicis brevis (APB) and intrinsic hand muscles with a relatively preserved abductor digiti mini muscle (ADM), a pattern classically known as a “split hand”, as well as slight weakness of the right arm (MRC, right/left): Deltoid 4/5, biceps 4/5, brachioradial 4/5, triceps 5-/5, wrist extension 5/5, finger extension 5/5, wrist flexion 5-/5, hand grip 5/5, APB 4/5. Deep tendon reflexes were now brisk in the upper limbs, and there were no signs of sensory impairment. Lower limbs were nearly completely paralyzed with an MRC grade of 0 in most proximal and distal muscles apart from left foot plantar and toe flexion (MRC grade 3). All deep tendon reflexes were absent; plantar response was flexor on both sides. Vibration sense was reduced (right/left 4/8 and 5/8), while the sensory deficit in the right lower extremity remained largely unchanged. Pulmonary work-up revealed a right diaphragmatic paresis. Sensory and motor neurographies were normal in the right ulnar nerve but unobtainable in the lower limbs. Motor evoked potentials to the upper extremities showed no abnormalities. Electromyography showed progressive signs of severe acute denervation in the upper limbs and the sternocleidomastoid muscle, with sparing of the tongue. A biopsy of the left superficial peroneal nerve revealed a reduction of myelinated fibers without signs of vasculitis. Serum NfL levels remained highly elevated and only slightly decreased at last follow-up [107.9 pg/ml; >99.9th percentile (see Figure 1)].

At this point, the clinical course was clearly deviating from expectance with deterioration under immunotherapy, brisk reflexes in wasted muscles, split hand, and split leg characteristics (20), bulbar involvement on myography and a nerve biopsy negative for vasculitis. This presentation allowed a diagnosis of laboratory supported probable ALS according to the El Escorial/Awaji criteria (2, 3) and a diagnosis of ALS according to the Gold Coast criteria (4) (see Table 1). The sensory involvement was most likely explained by coincidental, probably Sjögren's associated, sensory neuropathy.

Because of the patient's family history, genetic panel testing for motor neuron diseases was performed. It revealed a pathological repeat expansion in the C9*orf* 72 gene confirming a familial ALS. During the following months, the patient experienced a further decline in motor function and died from respiratory failure 26 months after symptom onset.

## Discussion

The pathological hallmark of ALS spectrum diseases is an abnormal nuclear clearance and development of cytoplasmatic inclusion of TDP-43 (21). Increased cortical excitability is thought to induce neurodegeneration through glutamate-mediated excitotoxicity. The predominant affection of monosynaptic projections may explain the differential involvement of muscle groups at the same joint resulting in the “split hand” or more generally, “split limb” phenotypes (20).

As there are no disease-specific biomarkers, the diagnosis of ALS is challenging and may be delayed due to comorbidities providing an alternative explanation of symptoms. However, a high level of diagnostic certainty should be attained, before confronting the patient with a diagnosis of a fatal disease without a curative treatment option, especially in cases in which the differential diagnosis is treatable.

The initial clinical picture presented by our patient was suggestive for a lesion in the proximal part of the peripheral nervous system involving motor and sensory fibers. Restriction to one side had suggested plexopathy, but asymmetric presentations of polyradiculopathy and mononeuritis multiplex were also considered. Extensive work-up revealed a Sjögren's syndrome. At that point in time, the monomelic presentation including sensory deficits was attributed to an assumed vasculitic neuropathy, where high levels of sNfL may also be found (13).

Neuropathies in Sjögren's syndrome can be axonal, demyelinating, and mixed, they predominantly involve sensory fibers with and without pain, but autonomous and motor fibers as well as cranial nerves may also be affected (23). Severe forms of motor neuropathy may even mimic ALS (17). Histopathologically, sensory neuropathies show an infiltration of dorsal root sensory ganglia, while motor neuropathies rather seem to be caused by vasculitis and micro-infarction of nerves (22).

Interestingly, an association of a variety of auto-inflammatory diseases with ALS has been described, among them rheumatologic diseases like Sjögren's syndrome (23). On the other hand, coincident sensory neuropathy may be found in ALS, in C9*orf* 72 ALS in up to 38% of subjects (24).

In our case, there are several hints, which retrospectively fit well with the final diagnosis. In particular, painless progressive spread of motor symptoms and a partly “split limb” phenotype are typical characteristics of ALS. The high concentrations of sNfL in the context of a nerve biopsy negative for vasculitis, absence of rapid progressive dementia and no other plausibleexplanation is a strong indicator for ALS as the likely cause of neuro-axonal damage. Furthermore, sNfL remained high despite immunosuppressive treatment while markers indicating activity of Sjögren's disease were low from the beginning. In the case of a vasculitic neuropathy, sNfL would have been expected to typically decrease when treated successfully (13), while in ALS levels usually remain stable over the disease course until the later stages (9, 25). These findings further substantiated doubts on a causative relationship between Sjögren's disease and the motor neuropathy. Nevertheless, UMN signs could only be found clinically several months later with brisk reflexes in wasted muscles. Studies of cortical excitability *via* transcranial magnetic stimulation as a candidate marker of UMN dysfunction early in the disease had not been performed. Imaging of brain and entire spinal cord showed only unspecific changes even late in the disease course.

Genetic testing at an earlier time point would have helped to shorten the diagnostic uncertainty, and the patient would not have been exposed to the risks and side effects of an immunosuppressive treatment. However, genetic testing is not yet done routinely. Furthermore, the C9*orf* 72 repeat expansion is intronic and only detectable by using repeat-primed PCR techniques, which are not included in common panel exome analysis and have to be ordered explicitly (26).

In conclusion, our case shows the difficulties of diagnosing ALS in the setting of a potentially relevant alternative diagnosis. Moreover, it highlights the need for diagnostic biomarkers as well as the role of genetic testing. These tools are important for development of disease-modifying therapies and for early diagnosis when future treatments may most efficaciously prevent disease progression.

## Data Availability Statement

The original contributions presented in the study are included in the article/supplementary material, further inquiries can be directed to the corresponding author/s.

## Ethics Statement

Ethical review and approval was not required for the study on human participants in accordance with the local legislation and institutional requirements. The patients/participants provided their written informed consent to participate in this study. Written informed consent was obtained from the individual(s) for the publication of any potentially identifiable images or data included in this article.

## Author Contributions

MJW: clinical and electrophysiological examinations and manuscript writing. JK: manuscript editing and analysis of serum biomarkers. MH: supervision of the clinical and examinations and supervision and editing of the writing process. All authors contributed to the article and approved the submitted version.

## Conflict of Interest

The authors declare that the research was conducted in the absence of any commercial or financial relationships that could be construed as a potential conflict of interest. The reviewer TR declared a past co-authorship with one of the authors JK to the handling editor.

## Publisher's Note

All claims expressed in this article are solely those of the authors and do not necessarily represent those of their affiliated organizations, or those of the publisher, the editors and the reviewers. Any product that may be evaluated in this article, or claim that may be made by its manufacturer, is not guaranteed or endorsed by the publisher.
